# Point-of-sale tobacco advertising in Beirut, Lebanon following a national advertising ban

**DOI:** 10.1186/1471-2458-13-534

**Published:** 2013-06-03

**Authors:** Ramzi G Salloum, Rima T Nakkash, Allison E Myers, Kathryn A Wood, Kurt M Ribisl

**Affiliations:** 1 Health Policy and Management, Gillings School of Global Public Health, University of North Carolina at Chapel Hill, 1102F McGavran-Greenberg, 135 Dauer Drive, Chapel Hill, NC 27599, USA; 2 Health Promotion and Community Health, Faculty of Health Sciences, American University of Beirut, Beirut, Lebanon; 3 Health Behavior, Gillings School of Global Public Health, University of North Carolina at Chapel Hill, Chapel Hill, NC, USA; 4 Duke University School of Nursing, Durham, NC, USA; 5 Health Behavior, Gillings School of Global Public Health, Lineberger Comprehensive Cancer Center, University of North Carolina at Chapel Hill, Chapel Hill, NC, USA

## Abstract

**Background:**

The objective of this study was to conduct an audit of point-of-sale (POS) tobacco advertising and assess compliance with an advertising ban in a large district of Beirut, Lebanon.

**Methods:**

The audit was conducted 3 months following the ban on tobacco advertising. Trained students observed all tobacco retail outlets (n = 100) and entered data into a web-based form using iPad® technology. Presence of tobacco advertisements was assessed to determine compliance with the national advertising ban.

**Results:**

Among the 100 tobacco retail outlets, 62% had tobacco advertisements, including 7% with a tobacco brand logo as part of the main exterior store sign.

**Conclusions:**

POS tobacco advertising is widespread in Beirut despite the national advertising ban. These findings point to an urgent need for the enforcement of the advertisement ban with tobacco retail outlets in Lebanon.

## Background

For decades, the multinational tobacco industry has enjoyed free and unrestricted tobacco product advertising, marketing, and sponsorship in Lebanon [1-3]. Analysis of tobacco industry documents has revealed that this laxness in regulation has been the outcome of a deliberate tobacco industry strategy to delay adoption and implementation and weaken the content of proposed regulation [4]. Smoking prevalence in Lebanon is estimated at 42.9% among adult males and 26.3% among adult females [5]; this is among the highest rates for females in the Middle East region. Among 13-15 year olds, 8.3% are current smokers (12.1% of boys and 5.6% of girls) [6] and there is an increase in evidence of tobacco advertising targeted to the Lebanese youth [1-4].

Lebanon ratified the Framework Convention on Tobacco Control (FCTC) in 2005 but it was not until 2011 that it adopted the first comprehensive tobacco control legislation. The law banned smoking in all indoor public places, effective September 2011. The ban became effective for the hospitality sector in September 2012. The legislation also banned all forms of advertising and sponsorship of tobacco products effective March 2012, and larger textual or pictorial warnings were dictated through the issuance of a ministerial decree.

Thus, for the first time in the country’s history, all forms of advertising and sponsorship of tobacco products became illegal. The Lebanese Ministry of Public Health, Ministry of Tourism, Ministry of Interior, and Ministry of Economy and Trade have been charged with enforcing the provisions of the new law [7]. In matters related to advertising, the Agency for Consumer Protection within the Ministry of Economy and Trade is responsible for enforcement. Violators of the advertising ban can face large fines of up to 40 million Lebanese Pounds (approximately $27,000 USD). A follow up Ministerial Decree No. 7437, issued in January 2012 [7], allowed retailers to display one sign inside their stores stating that tobacco products are sold on the premises, but places the following restrictions: (1) the sign can be no more than A5 in size (14.8 cm × 21 cm); (2) the text of the sign shall only read “Tobacco products are sold here”; and (3) no other logo and/or trademark is allowed except for that of the Lebanese Régie, the government authority with exclusive rights to import and export local tobacco products and issue licenses to tobacco growers.

To date, no studies have documented the prevalence of point-of-sale (POS) tobacco advertising in Lebanon or the Middle East region. Findings from a recent systematic review of store audit methods to capture tobacco products and marketing at POS [8] indicate only twelve studies to date that have assessed retailer compliance with a national regulation: these were in India [9,10], Mexico [11,12], the United Kingdom and Ireland [13-15], and the United States [16-20]. In general, these studies found lower compliance rates in developing countries (India and Mexico) and higher compliance rates in developed countries (example – up to 97% compliance immediately following implementing a law to remove POS tobacco displays in Ireland) [14].

The primary aim of our study was to assess compliance of tobacco retail outlets with a national ban on interior and exterior tobacco advertising in the *Ras Beirut* district of the Lebanese capital city. A secondary aim of the study was to document the number of exterior and interior tobacco advertisements and to survey tobacco product placement within stores. The store audits were performed 3 months after the law took effect.

## Methods

### Sample

The store audits were conducted in *Ras Beirut*, a diverse mixed-use district that occupies the northwestern quarter of the city and is home to the American University of Beirut. The research area comprises 10 city sectors, the majority of which have high urban density; printed maps of each sector were generated using Google Maps. The store audits were completed by 5 undergraduate students who received a half-day training session on how to create a census of the tobacco retail outlets and on how to complete the store audits. The training session included 10 “mock” audits of stores outside of our sampling area. The students canvassed the entire district with predetermined routes, marking the location of each store and assigning it a unique identification number, and thus creating a census of all tobacco retail outlets (*N* = 103) in the *Ras Beirut* district. With the exception of supermarkets, the students were able to identify the small tobacco retail outlets from the street because tobacco advertising and/or products were visible through the storefronts. We made the assumption that all supermarkets in the region sold tobacco products and thus the students were instructed to include them in their census of tobacco retail outlets. Next, the students conducted the interior and exterior audits and electronically recorded their observations. Prior to completing each interior audit, the students obtained permission from the store owner or clerk. Tobacco retail outlets observed were all stores that sold cigarettes including small, convenience stores or mini markets; tobacco or liquor stores; bakeries; gas stations; and large or super markets. A total of 100 audits were completed in June 2012 (3 stores did not sell cigarettes).

### Measures and data analysis

Store audit items were based on prior studies [16,21] and designed to solicit information on the number and placement of exterior and interior store advertisements and presence of Régie-compliant sign, and placement of tobacco products within 1 m of confectionery (candy) and cash registers. These items assessing amount of marketing and placement are commonly assessed in store audit studies [8].

Descriptive statistics were computed to characterize the quantity and nature of tobacco retail advertising. Data analyses were performed using SAS software (version 9.3, SAS Institute Inc., Cary, NC, USA).

## Results

**Figure 1:**
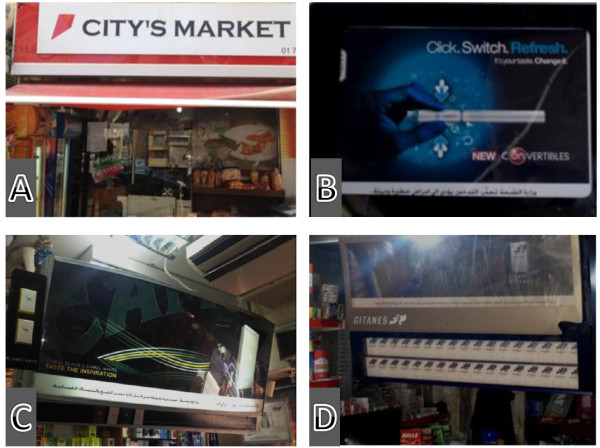
**Point-of-sale tobacco advertisement in *Ras Beirut *in June 2012, after decree that banned all tobacco product advertising.A**) Illegal brand stretching, exterior sign. **B**) Kent Switch Convertibles, interior sign. **C**) Camel, interior sign. **D**) Gitanes, interior sign.

Of the 100 tobacco retail outlets surveyed in *Ras Beirut*, 90% were small convenience stores or mini markets (Table 1). Overall, 62% of tobacco retail outlets had cigarette advertising. Exterior advertising was found in 14% of stores, half of which included cigarette brand logos as part of the main store sign (7 stores). Interior advertising was observed in 60% of stores. We did not observe exterior advertising in any of the large stores or supermarkets (results not shown). Overall, the advertisements we observed were limited to a few leading brands (Figure 1) including *Marlboro (Philip Morris)*, *Kent (British American Tobacco)*, *Camel (RJ Reynolds)*, *Davidoff and Gitanes (Imperial Tobacco)*. The Régie-compliant sign, required by Decree No. 7437, was not found in any of the audited stores. Tobacco products were placed within 1 m of candy in 81% of stores and within 1 m of the cash register in 98% of stores.

**Table 1:** **Point-of-sale tobacco audit results in *Ras Beirut *– June 2012**

	**Store audits (*n*** **= 100)**
Store type (%)	
Large store or supermarket	6
Small grocery or convenience store, mini market	90
Other	4
Point-of-sale advertising (%)	
Main exterior store sign contains tobacco logo	7
Stores with ≥ 1 exterior ad(s)	14
Average number of exterior ads	1.8
Stores with interior ads	
1-2	56
≥ 3	4
Average number of interior ads	1.4
Stores with ≥ 1 ad(s), including exterior and interior	62
Product placement within stores (%)	
Tobacco products < 1 m of candy	81
Tobacco products < 1 m of cash register	98

## Discussion

Our study results suggest a lack of compliance with the recent national tobacco advertising ban in Lebanon and urgently calls for enforcement of this law. Three months following the ban on tobacco advertising, 62% of stores in a large Beirut neighborhood were non-compliant with the new law. We found that tobacco advertisements were prevalent predominantly inside stores and to a smaller extent on exterior store signs and windows. Even though the new law clearly prohibits all forms of tobacco advertising – both outdoor and indoor – we observed more stores with interior advertising compared with exterior advertising. During our audits we observed empty sign holders especially on window fronts that had once displayed tobacco advertisements. Without longitudinal data, we cannot be certain that these advertisements were removed in response to, or directly following, the advertising ban.

On the other hand, we did not observe any retail outlets displaying the Régie-compliant sign. This was no surprise to us, given the low level of compliance with the advertising ban. Even among retailers compliant with the advertising ban, many may not be familiar with all provisions in the law and may be unaware of the particular decree related to displaying the sign. Our audit found that tobacco products are placed within 1 m of candy in 81% of stores and within 1 m of the cash register within 98% of stores. Placement of tobacco near confectionary is suggested to encourage adolescents to see tobacco as benign and commonplace.

Our study has strengths and limitations. This was the first study in Lebanon and the region to assess POS tobacco advertising and product placement within stores. Further, our study employed an innovative approach for data collection. The recent systematic review of POS audit methods did not find any published studies relying on electronic mobile input devices [8]. Although the relative accuracy of electronic input devices compared with paper/pencil has not been tested, we assume that it is as good as or better than paper because it does not involve transcribing data. A potential limitation of our study may be its cross-sectional design since we did not observe POS advertising prior to the recent ban. We assume that the advertising ban has been partially successful in reducing POS advertising. Based on our conversations with retailers, we learned that many had removed all advertising or relocated signs to less visible areas within stores in response to the new law. However we cannot report with certainty that advertising has declined without having a record of the POS advertising rate prior to the ban.

Faced with a comprehensive advertising ban in Lebanon, tobacco companies responded with illegal brand stretching practices [22]. Main exterior store signs with cigarette brand names have been replaced with signs that limit advertisement to brand colors and logos (Figure 1A). In many exterior and interior advertisements, the full *Kent* brand name and logo have been replaced with a simplified brand image, the “switch” logo to promote the *Kent Switch Convertibles* product, a cigarette which contains a liquid capsule in the filter that can be clicked to release flavor (Figure 1B). All 7 stores observed with advertising embedded into the store sign used the brand colors and logos in their advertisements.

Further, local news reports reveal that tobacco companies are persuading retailers to continue advertising under the pretext that the companies have challenged the new decree with the Ministry of Economy and Trade [23]. In their challenge of the decree, international tobacco companies replaced the term “public spaces” with “public streets” in an attempt to disable the outright and final legislation, which bans advertisements “in any manner that allows people passing in *public spaces* to see them.” The industry claimed that the ads “include information that enables consumers to make the right decision regarding consuming tobacco products and that taking them down constitutes an infringement on consumer protection law and obstructs consumers’ ability to discern between legal and illegal products” [23]. This kind of industry action is common in the face of advertising restrictions [24] and works to undermine the positive public health impact of such measures.

## Conclusions

Research on POS is important as it remains the least regulated channel of cigarette marketing and little is known on the potential impact of such regulation [24]. In addition to assessing compliance with the national advertising ban at points of sale, our study was the first in Lebanon to document tobacco product placement within stores. The new law did not address this issue. Similarly, our finding that the overwhelming majority of stores place cigarettes within 1 m of candy and cash registers calls for stricter regulation of tobacco promotion at point of sale.

## Competing interests

Dr. Ribisl and Ms. Myers have developed the Counter Tobacco Store Audit data collection system used in this study, and a tobacco retailer mapping system. Both will generate royalties when licensed. Dr. Ribisl is the Executive Director and Ms. Myers is the Deputy Director of Counter Tools, a nonprofit organization with a mission to disseminate store audit and mapping tools to public health practitioners working on policy change.

## Authors’ contributions

The study was jointly designed by all authors. KR and AM provided the data collection software/tool. RS, KW, and RN led the data collection. RS and AM were responsible for data analysis. Findings were jointly interpreted by all authors. All authors contributed to successive drafts. The final manuscript was approved by all authors.

## Pre-publication history

The pre-publication history for this paper can be accessed here:

http://www.biomedcentral.com/1471-2458/13/534/prepub
